# Supplementary material for: The skin microbiome facilitates adaptive tetrodotoxin production in poisonous newts
Source: eLife. 2020 Apr 7;9:e53898. doi: 10.7554/eLife.53898 (PMC7138609; doi:10.7554/eLife.53898)
Supplement: Figure 1—source data 1. — Estimated TTX concentrations (ng mL−1) were calculated relative to a calibration curve of pure TTX standards. [file elife-53898-fig1-data1.docx]

**Figure 1—source data 1:** Raw data for newt skin toxicity measurements by LC-MS/MS. Estimated TTX concentrations (ng mL^-1^) were calculated relative to a calibration curve of pure TTX standards.

| Newt ID | Location | Body mass (g) | Snout-vent length (mm) | Skin biopsy mass (mg) | Total TTX detected (ng mL^-1^) | Mass-adjusted TTX (ng mL^-1^) |
| --- | --- | --- | --- | --- | --- | --- |
| BC31 | Oregon | 22.9 | 81 | 2.5 | 351.6 | 140.6 |
| BC32 | Oregon | 24.3 | 79 | 1.2 | 187.1 | 155.9 |
| BC33 | Oregon | 25.9 | 79 | 3.0 | 82.0 | 27.3 |
| BC34 | Oregon | 23.2 | 82 | 1.7 | 444.8 | 261.6 |
| BC35 | Oregon | 20.3 | 79 | 1.2 | 56.4 | 47.0 |
| PF1 | Idaho | 15.8 | 70 | 3.0 | 0.0 | 0.0 |
| PF2 | Idaho | 16.3 | 69 | 3.2 | 0.0 | 0.0 |
| PF3 | Idaho | 20.5 | 79 | 9.0 | 0.0 | 0.0 |
| PF4 | Idaho | 17 | 75 | 7.0 | 0.0 | 0.0 |
| PF5 | Idaho | 12.3 | 69 | 5.6 | 0.0 | 0.0 |
| PF6 | Idaho | 9.3 | 66 | 2.0 | 0.0 | 0.0 |
| PF7 | Idaho | 15.6 | 76 | 4.0 | 0.0 | 0.0 |
| PF8 | Idaho | 13.4 | 75 | 1.8 | 0.0 | 0.0 |
| PF9 | Idaho | 12 | 77 | 2.0 | 0.0 | 0.0 |
| PF10 | Idaho | 12 | 70 | 1.0 | 0.0 | 0.0 |
| PF11 | Idaho | 9.4 | 67 | 1.5 | 0.0 | 0.0 |
| PF12 | Idaho | 17.4 | 74 | 2.8 | 0.0 | 0.0 |
| PF13 | Idaho | 20.6 | 84 | 1.0 | 0.0 | 0.0 |
| PF14 | Idaho | 16.2 | 82 | 5.5 | 0.0 | 0.0 |
| PF15 | Idaho | 14.2 | 80 | 1.2 | 0.0 | 0.0 |
| PF16 | Idaho | 13.9 | 74 | 4.0 | 0.0 | 0.0 |
| PF17 | Idaho | 15.4 | 76 | 3.6 | 0.0 | 0.0 |
