# Supplementary material for: The skin microbiome facilitates adaptive tetrodotoxin production in poisonous newts
Source: eLife. 2020 Apr 7;9:e53898. doi: 10.7554/eLife.53898 (PMC7138609; doi:10.7554/eLife.53898)
Supplement: Figure 1—source data 2. — The relative abundance of each bacterial OTU within a sample was calculated and averaged across all samples for each population; these data are shown below as percentages. Taxonomy was assigned using the Ribosomal Database Project (Cole et al., 2014) and a confidence threshold of 80%. OTUs shared between the two populations are in bold. [file elife-53898-fig1-data2.docx]

**Figure 1—source data 2:** The top 20 most abundant bacterial OTUs found among toxic (Oregon) and non-toxic (Idaho) newts. The relative abundance of each bacterial OTU within a sample was calculated and averaged across all samples for each population; these data are shown below as percentages. Taxonomy was assigned using the Ribosomal Database Project (Cole et al., 2014) and a confidence threshold of 80%. OTUs shared between the two populations are in bold.

| Population | OTU | Taxonomic Classification | Relative Abundance (%) |
| --- | --- | --- | --- |
| Oregon | 00002 | *Rhodoferax* | 19.45 |
|  | 00001 | Unclassified Flavobacteriaceae | 10.74 |
|  | 00003 | ***Rhodoferax*** | **6.85** |
|  | 00004 | **Unclassified Proteobacteria** | **3.49** |
|  | 00006 | Unclassified Verrucomicrobiales | 3.35 |
|  | 00005 | ***Romboutsia*** | **3.08** |
|  | 00022 | Unclassified Fusobacteriaceae | 2.59 |
|  | 00025 | Unclassified Bacteria | 2.42 |
|  | 00038 | *Arthrobacter* | 2.10 |
|  | 00007 | ***Clostridium* *sensu stricto*** | **2.06** |
|  | 00029 | Unclassified Bacteria | 1.66 |
|  | 00015 | ***Methylophilus*** | 1.16 |
|  | 00014 | **Unclassified Burkholderiales** | 1.53 |
|  | 00034 | *Flavobacterium* | 1.32 |
|  | 00008 | **Unclassified Bacteria** | 1.29 |
|  | 00017 | Unclassified SR1 | 1.23 |
|  | 00042 | *Pseudomonas* | 0.99 |
|  | 00011 | **Unclassified Comamonadaceae** | **0.88** |
|  | 00031 | Unclassified Peptostreptococcaeceae | 0.85 |
|  | 00027 | *Aeromonas* | 0.74 |
|  |  | Other genera | 32.22 |
|  |  |  |  |
| Idaho | 00004 | **Unclassified Proteobacteria** | **7.98** |
|  | 00003 | ***Rhodoferax*** | **6.55** |
|  | 00008 | **Unclassified Bacteria** | **5.25** |
|  | 00007 | ***Clostridium* *sensu stricto*** | **4.18** |
|  | 00012 | Unclassified Burkholderiales | 3.07 |
|  | 00010 | Unclassified Comamonadaceae | 2.97 |
|  | 00013 | Unclassified Betaproteobacteria | 2.70 |
|  | 00009 | Unclassified Methylococcaceae | 2.61 |
|  | 00005 | ***Romboutsia*** | **2.40** |
|  | 00011 | **Unclassified Comamonadaceae** | **2.24** |
|  | 00016 | Unclassified Burkholderiales | 1.66 |
|  | 00020 | Verrucomicrobiaceae | 1.52 |
|  | 00014 | **Unclassified Burkholderiales** | **1.43** |
|  | 00019 | Unclassified Sphingobacteriales | 1.33 |
|  | 00024 | Unclassified Methylococcaceae | 1.17 |
|  | 00015 | ***Methylophilus*** | **1.16** |
|  | 00023 | *Thiodictyon* | 1.09 |
|  | 00033 | Unclassified Opitutae | 1.03 |
|  | 00028 | *Rhodoferax* | 0.99 |
|  | 00018 | Unclassified Bacteria | 0.90 |
|  |  | Other genera | 47.77 |
