# Supplementary material for: The skin microbiome facilitates adaptive tetrodotoxin production in poisonous newts
Source: eLife. 2020 Apr 7;9:e53898. doi: 10.7554/eLife.53898 (PMC7138609; doi:10.7554/eLife.53898)
Supplement: Figure 1—source data 3. — Each value is shown as mean ± SEM. [file elife-53898-fig1-data3.docx]

**Figure 1—source data 3**: Variation in alpha diversity between newt populations and sampling sites across the bodies of individual newts. Each value is shown as mean ± SEM.

| Population | Body Site | Sample Size | Good’s Coverage | Number of OTUs | Chao1 Richness | Simpson Index (1-D) |
| --- | --- | --- | --- | --- | --- | --- |
| Oregon | Dorsal | 11 | 0.964 ± 0.013 | 397 ± 105 | 655 ± 214 | 0.921 ± 0.039 |
|  | Ventral | 10 | 0.965 ± 0.008 | 389 ± 85 | 634 ± 135 | 0.908 ± 0.038 |
|  | Cloaca | 12 | 0.968 ± 0.006 | 325 ± 65 | 593 ± 117 | 0.850 ± 0.035 |
|  | Chin | 8 | 0.967 ± 0.012 | 391 ± 132 | 612 ± 177 | 0.894 ± 0.066 |
|  |  |  |  |  |  |  |
| Idaho | Dorsal | 15 | 0.904 ± 0.019 | 828 ± 159 | 1766 ± 339 | 0.965 ± 0.018 |
|  | Ventral | 14 | 0.918 ± 0.019 | 697 ± 160 | 1509 ± 351 | 0.943 ± 0.027 |
|  | Cloaca | 13 | 0.922 ± 0.024 | 738 ± 196 | 1392 ± 424 | 0.957 ± 0.030 |
|  | Chin | 15 | 0.928 ± 0.022 | 635 ± 187 | 1283 ± 406 | 0.912 ± 0.031 |
