# Supplementary material for: The skin microbiome facilitates adaptive tetrodotoxin production in poisonous newts
Source: eLife. 2020 Apr 7;9:e53898. doi: 10.7554/eLife.53898 (PMC7138609; doi:10.7554/eLife.53898)
Supplement: Figure 4—source data 1. [file elife-53898-fig4-data1.docx]

**Figure 4—source data 1:** GenBank accession numbers of vertebrate Na_v_ channel protein sequences used in multiple sequence alignments and analysis.

| Protein | Species | GenBank accession number |
| --- | --- | --- |
| Na_v_1.1 | *Homo sapiens* | NM_001165963.2 |
|  | *Mus musculus* | NM_001313997.1 |
|  | *Gallus gallus* | XM_003641586.3 |
|  | *Anolis carolinensis* | XM_016995267.1 |
|  | *Nanorana parkeri* | XM_018554238.1 |
|  | *Xenopus tropicalis* | XM_012971277.2 |
| Na_v_1.2 | *Homo sapiens* | NM_021007.2 |
|  | *Mus musculus* | NM_001099298.3 |
|  | *Gallus gallus* | NM_001293281.1 |
|  | *Anolis carolinensis* | XM_008115152.2 |
|  | *Nanorana parkeri* | XM_018554236.1 |
|  | *Xenopus tropicalis* | XM_018097611.1 |
| Na_v_1.3 | *Homo sapiens* | NM_006922.3 |
|  | *Mus musculus* | NM_001355166.1 |
|  | *Gallus gallus* | XM_015289740.1 |
|  | *Anolis carolinensis* | XM_016995258.1 |
|  | *Nanorana parkeri* | XM_018554239.1 |
|  | *Xenopus tropicalis* | XM_012971260.2 |
| Na_v_1.4 | *Homo sapiens* | NM_000334.4 |
|  | *Mus musculus* | NM_133199.2 |
|  | *Gallus gallus* | NM_001318445.1 |
|  | *Anolis carolinensis* | XM_008113208.2 |
|  | *Nanorana parkeri* | XM_018560831.1 |
|  | *Xenopus tropicalis* | XM_018089322.1 |
|  | *Taricha granulosa* | KP118969.1 |
| Na_v_1.5 | *Homo sapiens* | NM_198056.2 |
|  | *Mus musculus* | NM_021544.4 |
|  | *Gallus gallus* | NM_001318446.1 |
|  | *Anolis carolinensis* | XM_016997604.1 |
|  | *Xenopus tropicalis* | XM_018094761.1 |
| Na_v_1.6 | *Homo sapiens* | NM_014191.3 |
|  | *Mus musculus* | NM_001077499.2 |
|  | *Gallus gallus* | XM_424477.4 |
|  | *Anolis carolinensis* | XM_008103947.2 |
|  | *Thamnophis sirtalis* | BK008864.1 |
|  | *Nanorana parkeri* | XM_018565420.1 |
|  | *Xenopus tropicalis* | NP_001361657 |
